# B Cell Homeostasis and Functional Properties Are Altered in an Hypochlorous Acid-Induced Murine Model of Systemic Sclerosis

**DOI:** 10.3389/fimmu.2017.00053

**Published:** 2017-02-07

**Authors:** Sébastien Sanges, Manel Jendoubi, Niloufar Kavian, Carine Hauspie, Silvia Speca, Jean-Charles Crave, Thomas Guerrier, Guillaume Lefèvre, Vincent Sobanski, Ariel Savina, Eric Hachulla, Pierre-Yves Hatron, Myriam Labalette, Frédéric Batteux, Sylvain Dubucquoi, David Launay

**Affiliations:** ^1^U995, LIRIC – Lille Inflammation Research International Center, Université de Lille, Lille, France; ^2^INSERM, U995, Lille, France; ^3^Département de Médecine Interne et Immunologie Clinique, CHU Lille, Lille, France; ^4^Centre National de Référence Maladies Systémiques et Auto-immunes Rares (Sclérodermie Systémique), Lille, France; ^5^Faculté de Médecine, Institut Cochin INSERM U1016 et Laboratoire d’immunologie biologique, AP-HP Hôpital Cochin, Université Paris Descartes, Sorbonne Paris-Cité, Paris, France; ^6^Institut d’Immunologie, CHU Lille, Lille, France; ^7^Octapharma France SAS, Medical Department, Boulogne-Billancourt, France; ^8^Institut Roche, Boulogne-Billancourt, France

**Keywords:** systemic sclerosis, B cell, regulatory B cells, interleukin-6, interleukin-10, animal model

## Abstract

**Introduction:**

During systemic sclerosis (SSc), peripheral B cells display alterations in subset homeostasis and functional properties and are a promising therapeutic target. However, there is only few data regarding whether these anomalies are accurately reproduced in animal models of SSc.

**Objective:**

In this work, we assessed the B cell homeostasis modifications in an experimental model of SSc [hypochlorous acid (HOCl)-induced mouse], both at a phenotypic and functional level, during the course of the disease.

**Methods:**

Balb/c mice underwent daily intradermal injections of HOCl (or phosphate-buffered saline) and were then sacrificed at day 21 (early inflammatory stage) or day 42 (late fibrotic stage). For phenotypic studies, the distribution of the main spleen cell subsets (B cells, T CD4 and CD8 cells, NK cells, macrophages) and splenic B cell subsets (immature, mature naïve, germinal center, antibody-secreting, memory, B1) was assessed by flow cytometry. For functional studies, splenic B cells were immediately MACS-sorted. Production of interleukin (IL)-6, CCL3, IL-10, and transforming growth factor (TGF)-β was assessed *ex vivo* by RT-PCR and after 48 h of culture by ELISA. Regulatory B cell (Breg) counts were quantified by flow cytometry.

**Results:**

Phenotypic analyses showed an early expansion of transitional B cells, followed by a late expansion of the mature naive subset and decrease in plasmablasts and memory B cells. These anomalies are similar to those encountered in SSc patients. Functional analyses revealed a B-cell overproduction of pro-inflammatory cytokines (IL-6 and CCL3) and an impairment of their anti-inflammatory capacities (decreased production of IL-10 and TGF-β, reduced levels of Bregs) at the early inflammatory stage; and an overproduction of pro-fibrotic cytokines (TGF-β and IL-6) at the late fibrotic stage. These results approximate the anomalies observed in human SSc.

**Conclusion:**

This work reports the existence of anomalies in B cell homeostasis and functional properties in an animal model of SSc that approximate those displayed by SSc patients. These anomalies vary over the course of the disease, which pleads for their participation in inflammatory and fibrotic events. This makes the HOCl mouse a relevant experimental model for the study of B cells, and therefore, B-cell-targeted therapies in SSc.

## Introduction

Systemic sclerosis (SSc) is a rare and severe condition classified within the connective tissue diseases. It is characterized by the progressive development of fibrosis in the skin and/or the internal organs such as lungs, digestive tract, and heart (1), impacting on vital and functional prognoses (2–5).

Systemic sclerosis pathophysiology is complex and only partially elucidated (6). It combines, to different degrees, a fibrotic (excessive synthesis of collagen fibers by activated fibroblasts), vascular (microangiopathy), and immunological (dysregulation of cellular and humoral immune systems) components. Among the different immunity actors involved in SSc, the almost-constant presence of autoantibodies and hypergammaglobulinemia has long suggested a potential implication of B cells in the pathogenesis of the disease (7).

Recent data reinforce this hypothesis. Several works have shown alterations in B cell homeostasis and function in SSc patients. For example, there is a decrease in circulating memory B cell counts and an increase in naive B cell counts (8–12). Other teams have documented an enhanced secretion of pro-inflammatory cytokines [such as interleukin (IL)-6], a reduced production of anti-inflammatory cytokines (such as IL-10), and a decrease in circulating regulatory B cell (Breg) counts (9–11, 13–15). In SSc patients, B cells have also been shown to induce pro-fibrotic characteristics in dermal fibroblasts (16). Finally, B cell-targeted therapeutic strategies like anti-CD20 antibodies have been suggested to be effective in this disease (13).

Overall, B cells appear to have an important role in SSc pathophysiology beyond the classical production of autoantibodies. A better description of B cell alterations in this disease, as well as animal models successfully recapitulating them, are therefore mandatory to further understand their pathogenic role and develop new therapeutic approaches. However, data on the modifications in B cell subset distribution and functional properties in animal models of SSc are scarce. Moreover, no study has ever considered a possible variation in B cell involvement during the course of SSc. This is of peculiar importance since several works have suggested that B cell-targeted therapeutic strategies may have different effects whether they are started at an early or late stage of the disease (17–20).

To address these issues, we used a novel murine model of SSc in which daily intradermal injections of hypochlorous acid (HOCl) induce a systemic fibrosis (21). We studied the modifications in B cell homeostasis, both at a phenotypic (B cell subsets distribution) and functional (cytokine production and interaction with fibroblasts) level, at different time points during the course of the experimental disease.

## Animals and Methods

### Induction of the Experimental Disease

#### Animals

Six-week-old female BALB/c mice (*Janvier Labs*) were used in all experiments. Animals were housed in a specific pathogen-free facility, within autoclaved ventilated cages with sterile food and water *ad libitum*, under constant room temperature and with 12-h day–night cycles. This study was carried out in accordance with the local and national guidelines (directive #68/609 CEE). The protocol was approved by the Regional Ethics Committee on Animal Experimentation.

#### Experimental Procedure

Experimental SSc was induced by daily intradermal injections of 300 µl of an HOCl-generating solution into the shaved backs of mice, using a 27-gauge needle and a 1-ml syringe, as previously described (21). The HOCl-generating solution was extemporaneously prepared by adding NaClO solution (9.6% as active chlorine) to a 100 mM KH_2_PO_4_ solution (pH 6.2). The NaClO amount was determined by measuring the optical density (OD) of the solution at 280 nm, and then adjusted to obtain an OD between 0.7 and 0.9. Control mice received injections of 300 µl of sterilized phosphate-buffered saline (PBS).

#### Sample Collection

Mice were sacrificed by cervical dislocation under deep CO_2_ anesthesia at either day 21 (early stage) or day 42 (late stage) after the first injection. All samples were collected at the time of euthanasia. Skin samples were collected near the injection site, and either immediately frozen and stored at −80°C (for biomolecular analyses), or fixed in a fresh 4% paraformaldehyde/PBS solution and embedded in paraffin (for histological and immunohistochemical analyses). Whole spleens were immediately dissected to create a spleen cell suspension, filtered on a 70-µm nylon mesh and depleted in erythrocytes using an appropriate lysing buffer (*Red Blood Cell Lysing Buffer Hybri-Max*, cat. #R7757, Sigma-Aldrich). Blood samples were collected by retro-orbital puncture, set aside for clotting for 30 min, then centrifuged, and stored at −80°C.

### Evaluation of the Experimental Disease

#### Measurement of Skin Fold Thickness

Skin fold thickness was assessed with a caliper by averaging the measure of two different locations near the injection site, twice a week until sacrifice.

#### Histological Evaluation

Skin samples embedded in paraffin were sliced into serial 4-µm sections. Dermal thickness at the injection site was assessed by performing a May-Grünwald–Giemsa (MGG) staining and measuring the distance between the epidermal–dermal junction and the dermal–subcutaneous fat junction at a 40-fold magnification using the ImageJ morphometic software (*U.S. National Institute of Health*) (22). Ten random measurements per section were performed by two blinded investigators and averaged for each section. Collagen deposition in the skin was evaluated by performing a picrosirius-red staining and delineating the stained area using a color deconvolution method (23). This allowed a precise quantification of the total area occupied by collagen deposits in each section. Expression of α-smooth muscle actin (α-SMA) in the skin was assessed by immunofluorescence with a specific anti-α-SMA antibody (clone 1A4, cat. #ab7817, Abcam) coupled with DAPI (1:1,000-fold dilution, Thermo Fischer Scientific). Non-specific binding was appreciated using an isotype control antibody. Protein visualization was performed by using a specific Alexa Fluor 488 goat anti-rabbit antibody.

#### Measurement of Hydroxyproline Content

Collagen content was assessed by using a colorimetric *Hydroxyprolin Kit Assay* (Sigma-Aldrich) according to the manufacturer’s protocol. Briefly, approximately 10 mg of skin were homogenized in 100 ml of water and hydrolyzed at 120°C for 3 h in an equal volume of concentrated hydrochloric acid (HCl, 12 M). Then, a colorimetric product, visualized at 560 nm and proportional to the hydroxyproline content, was generated by reaction of oxidized hydroxyproline in each sample with 4-(Dimethylamino)benzaldehyde.

#### Quantification of Fibrosis, Inflammation, and Proliferation Markers RNA Expression in Skin Samples

Approximately 0.5 cm of frozen skin samples were minced and mechanically homogenized. Then, total RNA was extracted with a *Nucleospin RNA kit* (Macherey-Nagel, Hoerdt, France) and eluted in RNAse-free water. The purity of RNA was evaluated by UV spectroscopy on a Nanodrop system from 220 to 350 nm. Then, 1 µg of total RNA was used to obtain single-stranded cDNA by using a specific *Reverse Transcription Kit* (Thermo Fisher Scientific) according to the manufacturer’s protocol. Quantitative RT-PCR was performed by using *LightCycler FastStart DNA Master SYBR Green I* (Thermo Fisher Scientific), according to the manufacturer’s protocol. Primers sets include TGFB for transforming growth factor (TGF)-β1, Acta2 for α-SMA, Fn1 for Fibronectin, COL1a1 for Collagen I-1III, Il-6 for IL-6, Il-1b for IL-1β, tnfa for tumor necrosis factor (TNF)-α, and Pcna for proliferating cell nuclear antigen (PCNA). Sequences and relative NCBI references for each gene are listed in Table S1 in Supplementary Material. All samples were amplified in duplicate. DNA quantification was expressed as critical threshold cycle (Ct) value, or rather the cycle number at which the DNA amplification was first detected. Relative gene expression value was calculated as *E* = 2^−^Δ^Ct^, where ΔCt is the difference in crossing points between GAPDH and each gene.

### Phenotypic Evaluation of B Cells

#### Flow Cytometry

Immediately after collection, spleen cells were counted using an automated method (*Flow-Count Fluorospheres*, cat. #7547053, Beckman Coulter); and their viability was assessed with propidium iodide staining. Approximately 1 million spleen cells were incubated with mouse anti-Fc receptor antibody (*FcBlock*, clone 2.4G2; cat. #553142, BD Biosciences) for 15 min at 4°C protected from light to limit non-specific antibody binding. They were next incubated with different antibody mixes for membrane staining (Table S2 in Supplementary Material) for 20 min at 4°C protected from light. Data were then acquired on a 3-laser cytometer (*Navios*, Beckman Coulter) and analyzed with a dedicated software (*Kaluza*, Beckman Coulter).

#### Quantification of Serum B Cell-Activating Factor (BAFF) Levels

B cell-activating factor levels were assessed on serum samples in duplicate at a 1:3-dilution using an ELISA assay (*Quantikine ELISA Mouse BAFF*, cat. #MBLYS0, R&D systems), according to the manufacturer’s protocol.

#### Quantification of CD19 Expression in Skin Samples

CD19 expression in skin samples was assessed by immunofluorescence using a specific anti-CD19 antibody (0.5 µg/ml, BioLegend) overnight at 4°C, coupled with DAPI (1:1,000-fold dilution, Thermo Fischer Scientific). Protein visualization was performed by using a specific Alexa Fluor 566 goat anti-mouse antibody incubated for 1 h at room temperature.

### Functional Evaluation of B Cells

#### B Cell Sorting

Immediately after collection, B cells were isolated from splenocytes using a negative magnetic bead-assisted sorting assay (*EasySep Mouse Pan-B Cell Isolation Kit*, cat. #19844, StemCell), according to the manufacturer’s protocol. B cell purity was always above 95% as assessed by flow cytometry.

#### Quantification of IL-6, CCL3, IL-10, and TGF-β RNA Expression in B Cells

Immediately after sorting, purified B cells were ultra-centrifuged, treated with lysis buffer (*NucleoSpin RNA kit*, Macherey-Nagel), and stored at −80°C. Quantification of IL-6, chemokine ligand (CCL) 3, IL-10, and TGF-β RNA expression in B cells was performed as detailed in Section “Quantification of Fibrosis, Inflammation, and Proliferation Markers RNA Expression in Skin Samples” (except that levels were normalized to GUSB).

#### Quantification of IL-6, IL-10, and CCL3 Protein Levels in B Cells

Immediately after sorting, purified B cells were seeded on a 96-well plate (400,000 B cells per well) within complete medium [Roswell Park Memorial Institute (RPMI) 1640 medium containing 10% heat-inactivated fetal calf serum (FCS), 20 UI/ml penicillin, 20 µg/ml streptomycin, 2 mM l-glutamin, 1 mM pyruvate, 50 µM 2-mercaptoethanol, and 1% non-essential amino acids]. They were cultured during 48 h at 37°C in humidified atmosphere with 5% CO_2_ and stimulated either with lipopolysaccharide (LPS) (*Escherichia coli* serotype O127:B8, 10 µg/ml; cat. #L4516, Sigma-Aldrich), with LPS and anti-CD40 antibody (clone HM40/3, 2.5 µg/ml; cat. #553721, BD Biosciences), or without immunostimulation. After culture, supernatants were collected and immediately stored at −80°C.

Interleukin-6, IL-10, and CCL3 protein levels in supernatant samples were assessed in duplicate using ELISA assays (*Quantikine ELISA Mouse IL-6*, cat. #M6000B; *Quantikine ELISA Mouse IL-10*, cat. #M1000B; *Quantikine ELISA Mouse CCL3*, cat. #MMA00; R&D systems), at appropriate dilutions (1:10 for IL-6; 1:1 for IL-10; 1:100 for CCL3). All experiments were conducted according to the manufacturer’s protocols.

#### IL-10 Intracellular Staining

Immediately after sorting, purified B cells were seeded on a 48-well plate (2 million B cells per well) within complete medium (RPMI 1640 medium containing 10% heat-inactivated FCS, 20 UI/ml penicillin, 20 µg/ml streptomycin, 2 mM l-glutamin, 1 mM pyruvate, 50 µM 2-mercaptoethanol, and 1% non-essential amino-acids). As previously described (24), they were cultured during 48 h at 37°C in humidified atmosphere with 5% CO_2_ and stimulated by anti-CD40 antibody (clone HM40/3, 1 µg/ml; cat. #553721, BD Biosciences). During the last 5 h of culture, LPS (*E. coli* serotype O111:B4, 10 µg/ml; cat. #L4391, Sigma-Aldrich), PMA (50 ng/ml, cat. #P8139, Sigma-Aldrich), ionomycin (500 ng/ml, cat. #I0634, Sigma-Aldrich), and monensin (2 mM, cat. #00-4505-51, eBiosciences) were added to the culture medium to induce IL-10 expression and block exocytosis (24).

Interleukin-10 intracellular detection was performed as previously described (24). First, B cells were stained with a viability dye (*Live/Dead Fixable Far Red Dead Cell Stain Kit*, 1:3,200-dilution, cat. #L10120, Life Technologies), saturated with an anti-Fc receptor antibody (*FcBlock*, clone 2.4G2; cat. #553142, BD Biosciences) to prevent non-specific binding, and stained with an anti-CD19 antibody (*Brillant Violet 510 Rat Anti-Mouse CD19*, clone 1D3; cat. # 562956, BD Biosciences). Next they were fixed and permeabilized with the *Cytofix/Cytoperm* kit (cat. #554722, BD Biosciences) according to the manufacturer’s protocol. Permeabilized cells were then stained with an anti-IL-10 antibody (*PE Rat Anti-Mouse IL-10*, clone JES5-16E3; cat. #554467, BD Biosciences). Data were acquired on a 3-laser cytometer (*Navios*, Beckman Coulter) and analyzed with a dedicated software (*Kaluza*, Beckman Coulter).

#### Fibroblasts–B Cells Coculture Experiments

Mouse embryonic fibroblast cells (BALB/c 3T3, ATCC clone A31) were purchased from American Type Culture Collection (ATCC; MD, USA). B cells were sorted as described in Section “B Cell Sorting.” Cells were cultured in complete medium (RPMI 1640 medium containing 10% heat-inactivated FCS, 20 UI/ml penicillin, 20 µg/ml streptomycin). 3T3 fibroblasts (10^5^ cells) alone, B cells (5 × 10^5^ cells) alone, or cocultures were seeded in 12-well plates for 72 h.

Quantification of fibrosis and proliferation markers RNA expression in fibroblasts was performed as detailed in Section “Quantification of Fibrosis, Inflammation, and Proliferation Markers RNA Expression in Skin Samples.”

### Statistical Analysis

Multi-group comparisons were performed using Kruskall and Wallis non-parametric test. Whenever a significant difference was detected, Mann–Whitney test was used to compare each group by pair. All statistical analyses were performed on SPSS v22 (IBM) and GraphPad Prism v6 (GraphPad Software). Quantitative data were expressed as mean ± SEM. Significance was set at *p* < 0.05. In figures, levels of significance were pictured as follows: ****p ≤ 0.0001; ***p ≤ 0.001, **p ≤ 0.01, *p ≤ 0.05, °*p* ≤ 0.20; and ns for *p* ≥ 0.20.

## Results

### HOCl Injections Induce an Experimental Disease Whose Characteristics and Course Resemble Human SSc

#### HOCl Intradermal Injections Induce Skin Fibrosis in Mice

**Figure 1:**
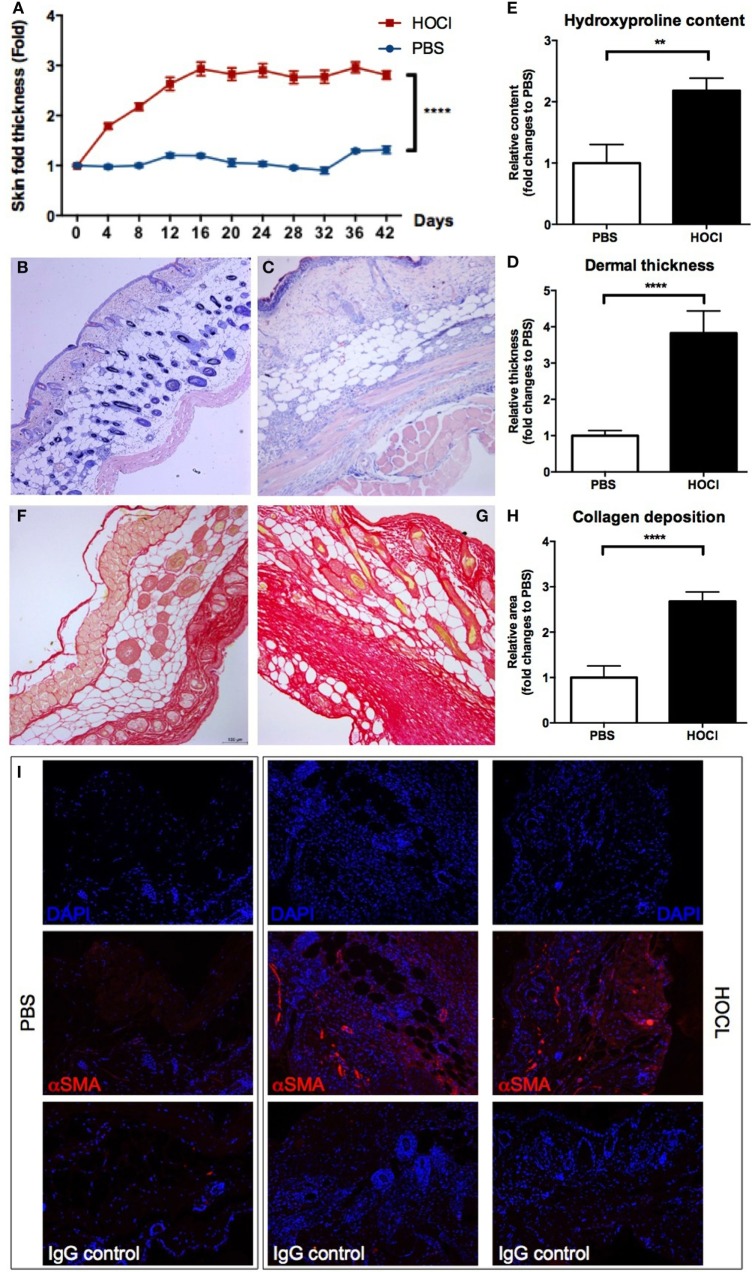
**Evaluation of skin fibrosis in phosphate-buffered saline (PBS) and HOCl mice**. To confirm the proper induction of the experimental disease, skin fibrosis was assessed by different methods in PBS and HOCl mice at day 42 after the beginning of the protocol. **(A)** Skin fold thickness (mm) measured sequentially by caliper (*n* = 20–26 per group). **(B,C)** Representative images of skin sections stained with May-Grünwald–Giemsa coloration from PBS [**(B)**; 10×] and HOCl [**(C)**; 10×] mice, showing dermal thickening in the HOCl mouse. **(D)** Dermal thickness measured on skin sections (*n* = 10–12 per group). Results are expressed as fold changes compared to the PBS group. **(E)** Hydroxyproline content in 6-mm skin punch biopsies (*n* = 7–8 per group). Results are expressed as fold changes compared to the PBS group. **(F,G)** Representative images of skin sections stained with picrosirius-red coloration from PBS [**(F)**; 10×] and HOCl [**(G)**; 10×] mice, showing collagen deposition in the HOCl mouse. **(H)** Collagen deposition total surface measured on skin sections (*n* = 10–12 per group). Results are expressed as fold changes compared to the PBS group. **(I)** Representative images of skin sections immunostained with DAPI alone (top row), DAPI and anti-α-smooth muscle actin (α-SMA) antibody (middle row), or DAPI and isotype control (bottom row) from PBS (left column) and HOCl (center and right columns) mice, showing expression of α-SMA in the HOCl mouse.

In a first step, we wanted to confirm the validity of the experimental disease. Induction of skin fibrosis in HOCl mice was assessed through clinical, protein, and histological evaluations (Figure 1). Thus, we observed a significant increase in the macroscopic skin fold in HOCl mice compared to PBS mice (*p* < 0.0001) (Figure 1A). Dermal thickness, measured on MGG-stained skin sections, was also significantly higher in the HOCl group than in the control group (*p* < 0.0001) (Figures 1B–D). A higher amount of collagen fibers in skin sections of HOCl mice, compared to PBS mice, was observed by histological picrosirius-red staining (*p* < 0.0001) and confirmed by evaluation of the hydroxyproline content, which was significantly increased in HOCl mice (*p* < *0.05*) (Figures 1E–H). Finally, a consistent amount of α-SMA-positive cells on skin from HOCl mice was qualitatively appreciated by immunofluorescence, whereas PBS mice showed a very low number of α-SMA-expressing cells (Figure 1I). Overall, these results indicated the validity of the experimental disease, as previously described (21).

#### The Early Stage of the HOCl Model Displays More Inflammatory Features than the Late Stage

As human SSc usually evolves through an early inflammatory stage and a late fibrotic stage, we wondered whether a similar phenomenon occurred during the course of the HOCl experimental disease. An evaluation of mRNA expression profile of various fibrosis (fibronectin, α-SMA, collagen, TGF-β), inflammatory (TNF-α, IL-1β, IL-6), and proliferation (PCNA) markers in the skin of PBS and HOCl mice was thus performed at two different time points from the beginning of the protocol (day 21: early stage; day 42: late stage).

**Figure 2:**
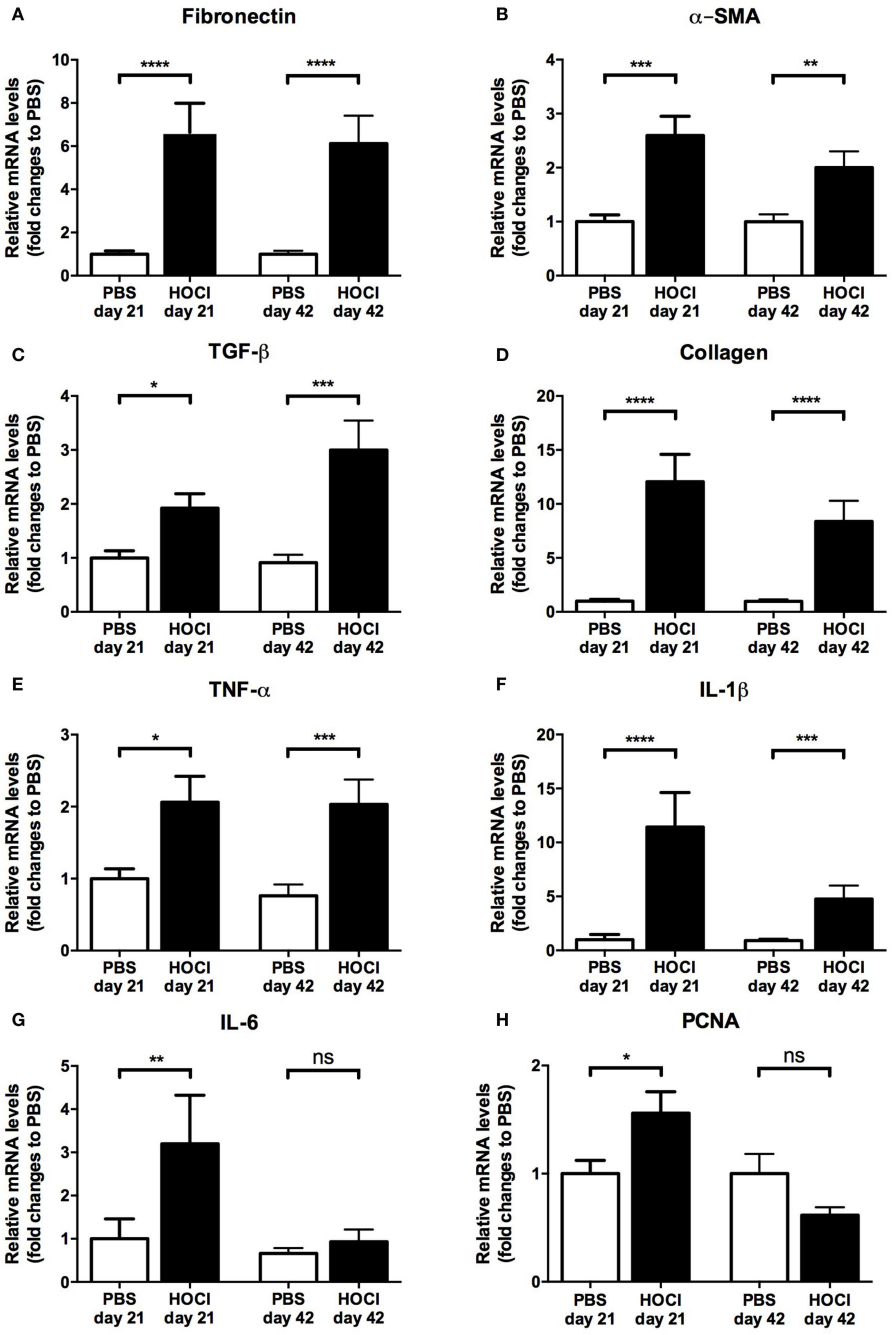
**Expression of fibrosis, inflammation, and proliferation markers in the skin of phosphate-buffered saline (PBS) and HOCl mice at day 21 and day 42 of the protocol**. mRNA levels of various markers of fibrosis [fibronectin **(A)**, α-smooth muscle actin (α-SMA) **(B)**, transforming growth factor (TGF)-β **(C)**, collagen **(D)**], inflammation [tumor necrosis factor (TNF)-α **(E)**, interleukin (IL)-1β **(F)**, IL-6 **(G)**], and proliferation [proliferating cell nuclear antigen (PCNA) **(H)**], normalized to GAPDH and expressed as fold changes to PBS day 21 (for day-21 groups) or to PBS day 42 (for day-42 groups) (*n* = 15–24 per group).

At day 21, we observed a significant increase in the mRNA expression of fibrotic, inflammatory, and proliferation markers in the HOCl mice compared to PBS mice (fibronectin: *p* < 0.0001; α-SMA: *p* = 0.0004; collagen: *p* < 0.0001; TGF-β: *p* = 0.01; TNF-α: *p* = 0.02; IL-1β: *p* < 0.0001; IL-6: *p* = 0.004; PCNA: *p* = 0.04) (Figure 2). At day 42, a significant increase in the mRNA levels of all pro-fibrotic proteins was still observed (fibronectin: *p* < 0.0001; α-SMA: *p* = 0.001; collagen: *p* < 0.0001; TGF-β: *p* = 0.0001). However, among inflammatory and proliferation markers, only TNF-α and IL-1β were differentially expressed (*p* = 0.0006 and *p* = 0.0005, respectively); there was no longer a significant difference in the production of IL-6 (*p* = 0.67) and PCNA (*p* = 0.31) (Figure 2). Overall, these results suggested that the early stage of the experimental disease displays more inflammatory and proliferative features than the late stage, which mimics to some extent the natural course of human SSc.

### Splenic B Cell Homeostasis Is Altered in HOCl Mice

#### Splenic B Cell Subset Distribution Is Modified in HOCl Mice and Varies over Time

**Figure 3:**
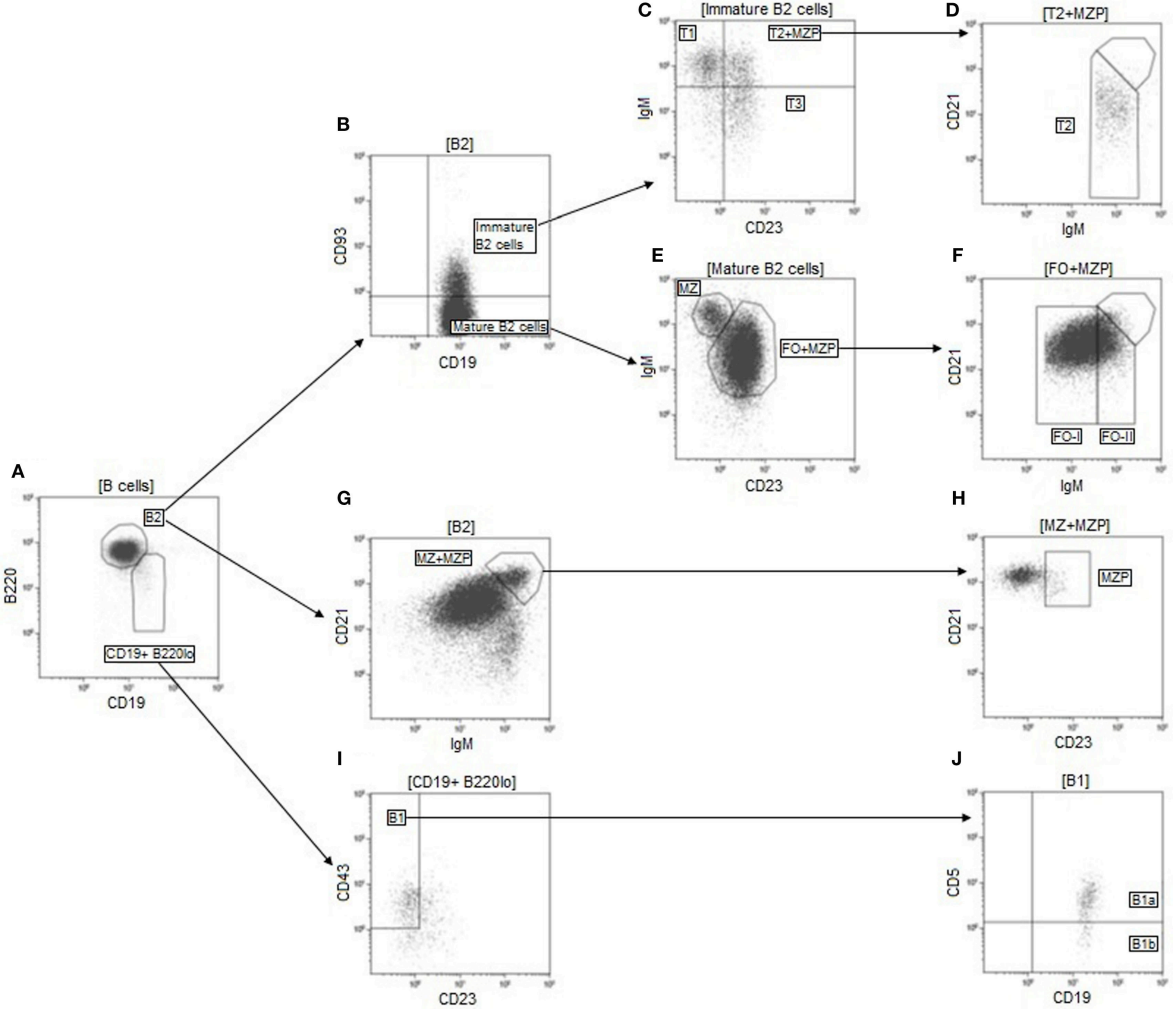
**Gating strategy used for identification of the main B cell subsets**. Doublets were first excluded on an FSC-area/FSC-height dot-plot (not shown). Lymphocytes were then selected based on their size and internal complexity on a FSC/SSC dot-plot (not shown). B cells were next delimited on a CD19/open channel dot-plot and defined as CD19^+^ non-autofluorescent cells (not shown). B2 cells were identified as CD19^+^ B220^hi^ cells on a CD19/B220 dot-plot gated on B cells **(A)**. Transitional B cells were then selected as CD93^+^ cells **(B)** and divided into their three main subsets based on their expression of CD23 and IgM **(C,D)**. Among mature B2 cells, defined as CD93^−^ cells **(B)**, follicular (FO) and marginal zone (MZ) B cells were separated based on their membrane levels of CD23 and IgM **(E)**. FO B cells were further divided into follicular type I (FO-I) and follicular type II (FO-II) depending on their expression levels of IgM **(F)**. MZP B cells were identified as CD23^hi^ cells within a gate containing CD21^hi^ IgM^hi^ B2 cells **(G,H)**. MZP cells were then backgated out of the T2 and FO populations **(D,F)**. B1 cells were identified as CD23^−^ CD43^+^ cells among CD19^+^ B220^hi^ cells **(A,I)**. B1a cells were then separated from B1b cells based on their membrane expression of CD5 **(J)**. FSC, forward scatter; Ig, immunoglobulin; SSC, side scatter.

Several works have reported disturbances in B cell homeostasis in SSc, especially in B cell subset distribution. We wondered if such anomalies existed in the HOCl model, and if so, how they would evolve during the course of the experimental disease. We compared the main leukocyte and B cell subsets in the spleens of HOCl and PBS mice, at the early and late stages of the disease. The phenotypic definitions and gating strategy used for their identification are detailed in Table 1 and Figure 3, respectively.

**Table 1:** **Phenotypic definitions of spleen cell and B cell subsets**.

Cell subsets	Abbreviation	Phenotypic definition
**Spleen cell subsets**		
**B cell**	/	CD19^+^
**T cell**	/	CD3^+^
T CD4^+^ cell	/	CD3^+^ CD4^+^
T CD8^+^ cell	/	CD3^+^ CD8^+^
**NK cell**	/	CD335^+^
**Monocyte–macrophage**	MM	CD11b^+^ FSC^hi^
**B cell subsets**		
**B2 cell**	B2	CD19^+^ B220^hi^
**Immature B cells**		
=Transitional B cell	TR	CD19^+^ B220^hi^ CD93^+^
Type 1 transitional B cell	T1	CD19^+^ B220^hi^ CD93^+^ CD23^−^ IgM^hi^
Type 2 transitional B cell	T2	CD19^+^ B220^hi^ CD93^+^ CD23^+^ IgM^hi^
Type 3 transitional B cell	T3	CD19^+^ B220^hi^ CD93^+^ CD23^+^ IgM^lo^
Marginal zone precursor B cell	MZP	CD19^+^ B220^hi^ CD93^−/+^ CD23^hi^ IgM^hi^ CD21^hi^
**Mature naive B cells**		CD19^+^ B220^hi^ CD93^−^
Follicular B cell	FO	CD19^+^ B220^hi^ CD93^−^ CD23^+^ CD21^lo/med^
Follicular type I B cell	FO-I	CD19^+^ B220^hi^ CD93^−^ CD23^+^ CD21^lo/med^ IgM^lo^
Follicular type II B cell	FO-II	CD19^+^ B220^hi^ CD93^−^ CD23^+^ CD21^lo/med^ IgM^hi^
Marginal zone B cell	MZ	CD19^+^ B220^hi^ CD93^−^ CD23^−^ CD21^hi^ IgM^hi^
**Nature non-naïve B cells**		
Germinal center B cells	GC	CD19^+^ CD38^−^ GL7^+^
Centroblasts	CB	CD19^+^ CD38^−^ GL7^+^ CXCR4^+^
Centrocytes	CC	CD19^+^ CD38^−^ GL7^+^ CXCR4^−^
Antibody-secreting cells	ASC	CD138^hi^ CD19^lo/−^
Plasmablasts	PB	CD138^hi^ CD19^lo^ CD22^+^
Plasma cells	PC	CD138^hi^ CD19^lo/−^ CD22^−^
Memory B cells	MemB	CD19^+^ CD93^−^ CD38^+^ IgM^−^
**B1 cells**	B1	CD19^+^ B220^lo^ CD23^−^ CD43^+^ IgM^hi^
B1a B cells	B1a	CD19^+^ B220^lo^ CD23^−^ CD43^+^ IgM^hi^ CD5^+^
B1b B cells	B1b	CD19^+^ B220^lo^ CD23^−^ CD43^+^ IgM^hi^ CD5^−^
**Regulatory B cells**		
CD5^+^ CD1d^hi^ B cells	/	CD19^+^ CD5^+^ CD1d^hi^
IL-10^+^ B cells	B10	CD19^+^ IL-10^+^

*CXCR, CXC-chemokine receptor; FSC, forward scatter; Ig, immunoglobulin; IL, interleukin*.

**Figure 4:**
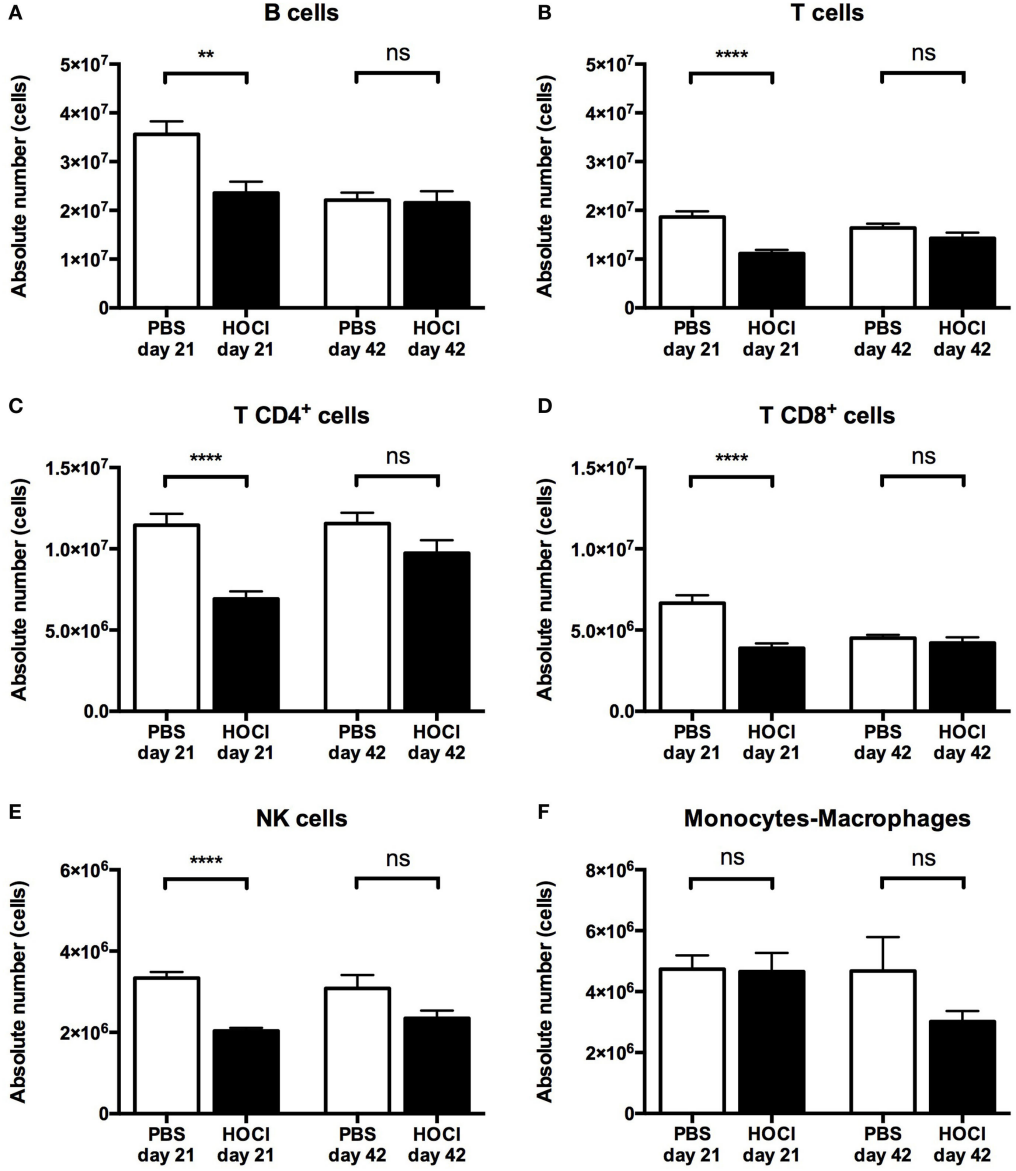
**Distribution of the main spleen cell subsets in phosphate-buffered saline (PBS) and HOCl mice at day 21 and day 42**. Distribution of B cells **(A)**, T cells **(B)**, T CD4^+^ cells **(C)**, T CD8^+^ cells **(D)**, NK cells **(E)**, and monocytes–macrophages **(F)** in absolute values expressed in cells (*n* = 12–17 per group).

Regarding total splenic B cell counts, we observed a significant decrease in HOCl mice at day 21 (*p* = 0.007), but no difference between the two groups at day 42 (*p* = 0.53) (Figure 4A). A similar trend was noted for the other splenic lymphocyte subsets, with a significant reduction in T CD4^+^, T CD8^+^, and NK cells at the early stage, and no difference at the late stage (Figures 4B–E). Monocyte and macrophage counts were equivalent at both stages of the disease (Figure 4F).

**Figure 5:**
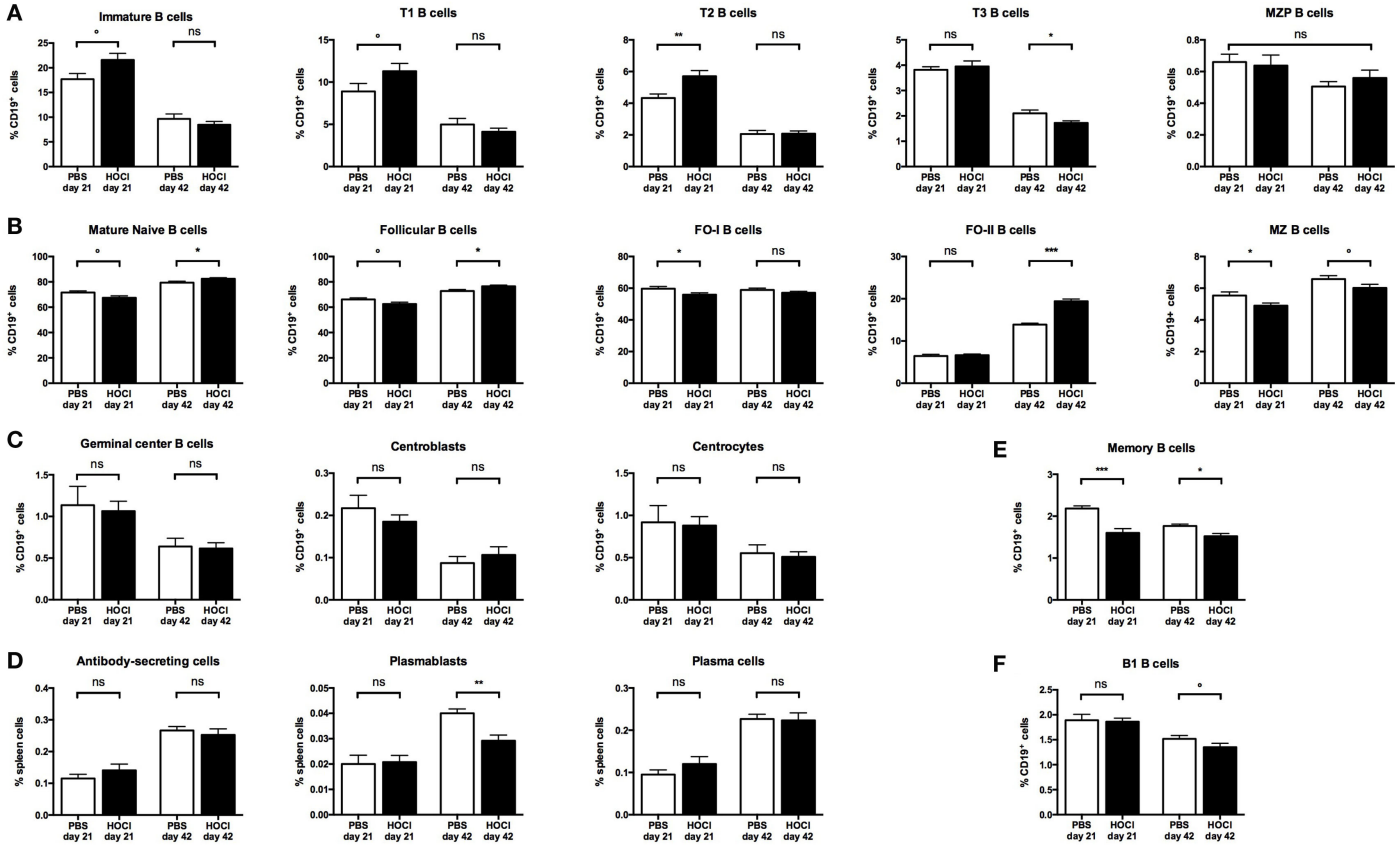
**Distribution of the main B cell subsets in phosphate-buffered saline (PBS) and HOCl mice at day 21 and day 42**. Distribution of immature B cells and its main subsets **(A)**, mature naive B cells and its main subsets **(B)**, germinal center B cells and its main subsets **(C)**, antibody-secreting cells and its main subsets **(D)**, memory B cells **(E)**, and B1 cells **(F)** in relative values expressed in percentages of CD19^+^ cells (except for ASC, PB, and PC expressed in percentages of total spleen cells) (*n* = 7–8 per group).

**Figure 6:**
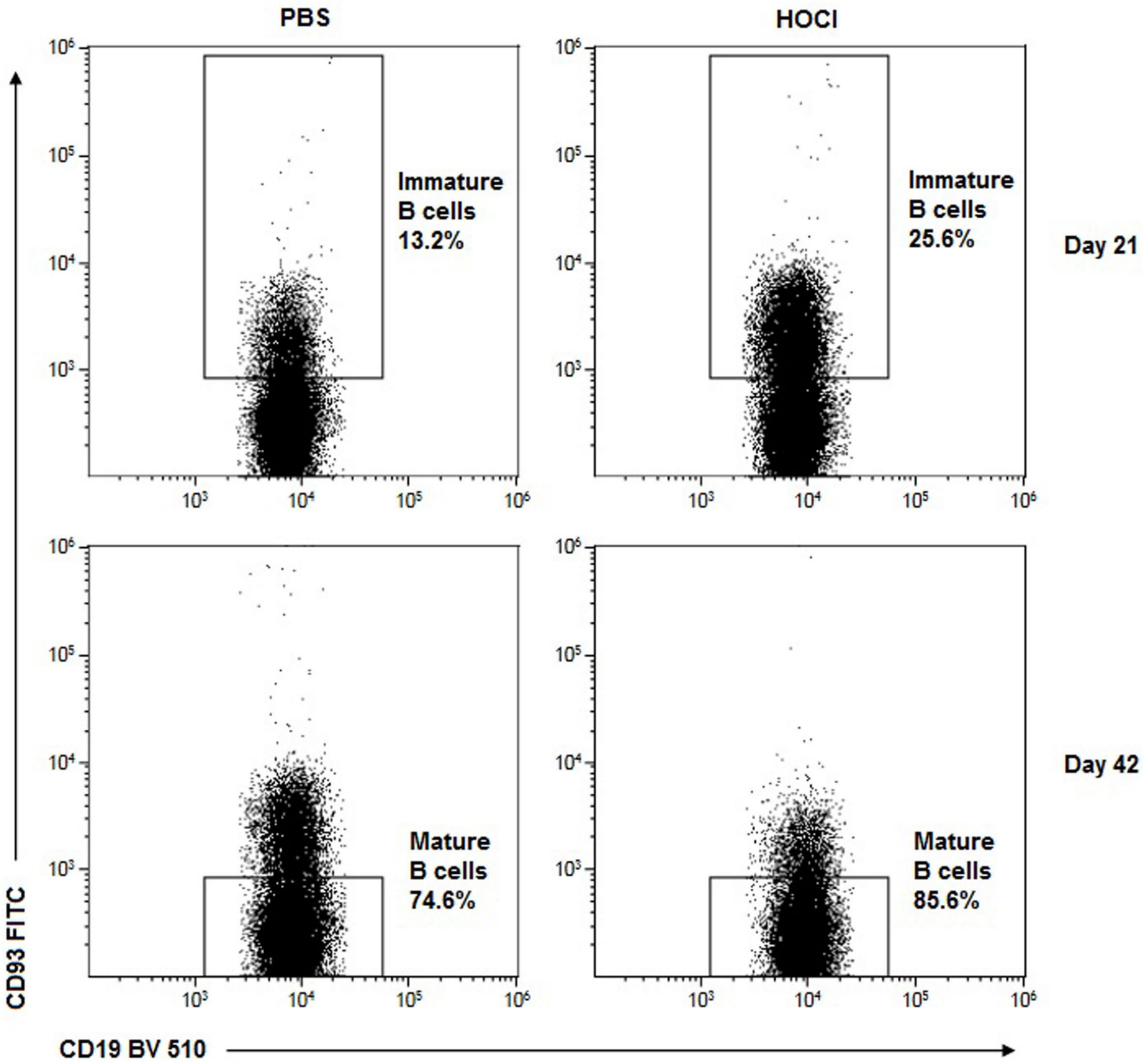
**Representative dot-plots of immature and mature B2 cells in phosphate-buffered saline (PBS) and HOCl mice at day 21 and day 42**. Representative CD19/CD93 dot-plots gated on B cells from PBS (left column) and HOCl (right column) mice at day 21 (top row) and day 42 (bottom row), showing early expansion of the transitional subset and late expansion of the mature naïve cells in the HOCl mouse.

Regarding immature B cell subsets (Figures 5A and 6), there was a trend for an increase in transitional forms at day 21 (*p* = 0.07) and a non-significant decrease at day 42 (*p* = 0.33) in HOCl mice. These anomalies reached statistical significance for transitional type 2 (T2) B cells at the early stage (*p* = 0.009) and transitional type 3 (T3) B cells at the late stage (*p* = 0.03).

Regarding mature naive subsets (Figures 5B and 6), the opposite phenomenon was noted, with a trend for a decrease at day 21 (*p* = 0.07) and a significant increase (*p* = 0.04) at day 42. These anomalies were mainly carried by the major mature naive subset, called follicular (FO) B cells (*p* = 0.07 at day 21; *p* = 0.02 at day 42). Interestingly though, the early FO decrease was essentially explained by the follicular type I B cells (*p* = 0.05) whereas the late FO increase was mostly supported by the follicular type II B cells (*p* = 0.0003). Conversely, the minor mature naive subset, named marginal zone B cells, was non-significantly decreased at both stages of the disease (*p* = 0.05 at day 21; *p* = 0.10 at day 42).

Regarding non-naive B cell subsets, germinal center B cell counts were similar in both groups at both time points, even when considering the centroblast and centrocyte subsets (Figure 5C). Although there was no difference in antibody-secreting cell and plasma cell counts between the two groups, a significant decrease in the plasmablast subset was noted at day 42 (*p* = 0.003) (Figure 5D). Switched memory B cells were also decreased in HOCl mice at both stages of the disease (*p* = 0.0007 at day 21; *p* = 0.001 at day 42) (Figure 5E).

Regarding B1 B cells (Figure 5F), we observed a trend for a decrease in HOCl mice at day 42 (*p* = 0.09) that reached significance for the B1a subset (*p* = 0.04).

Overall, these results confirmed that the HOCl model displays alterations in splenic B cell subset distribution (early expansion of transitional B cells; late expansion of the mature naive subset; and decrease in plasmablasts and memory B cells).

#### Alterations in B Cell Subset Homeostasis Are Independent of BAFF

**Figure 7:**
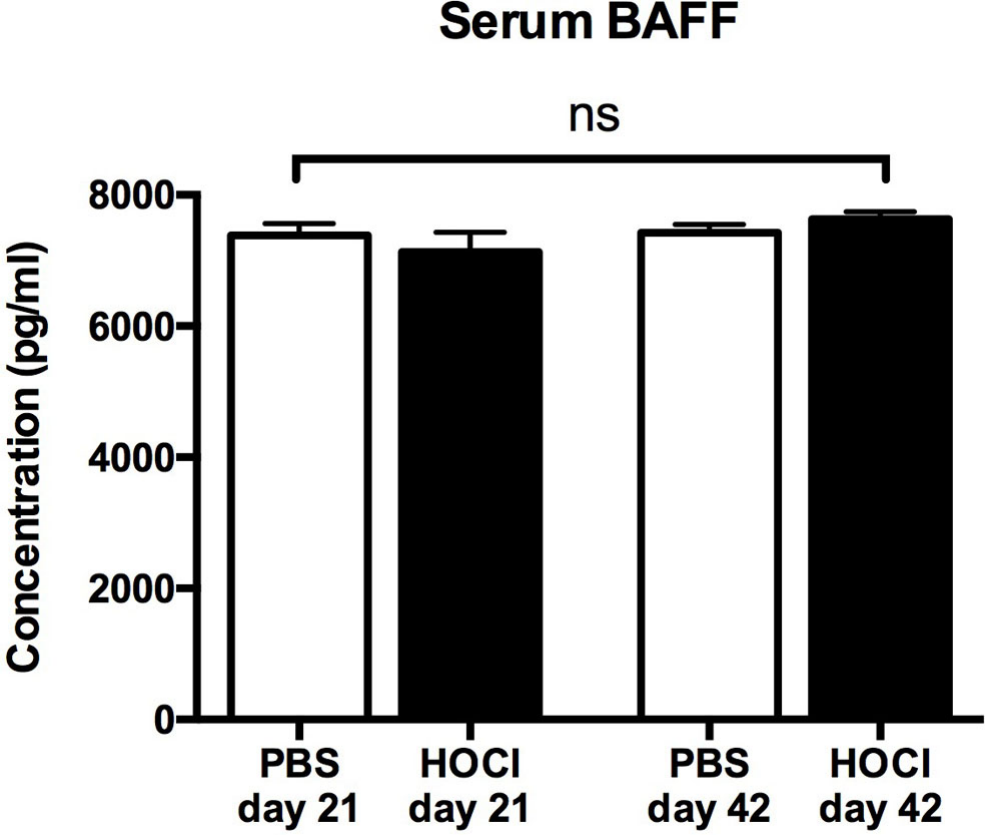
**Serum B cell-activating factor (BAFF) levels in phosphate-buffered saline (PBS) and HOCl mice at day 21 and day 42**. Results expressed in picograms per milliliter (*n* = 6–7 per group).

B cell homeostasis and maturation are regulated by a large number of factors among which survival signals (such as BAFF) play a critical role. Since serum BAFF levels are increased in SSc patients, we wondered if it could contribute to the alterations in B cell subset distribution observed in the HOCl model. However, serum BAFF levels were identical in both groups at both time points (Figure 7), suggesting that this cytokine does not participate in these anomalies.

#### HOCl Skin Is Infiltrated by B Cells at the Late Stage

**Figure 8:**
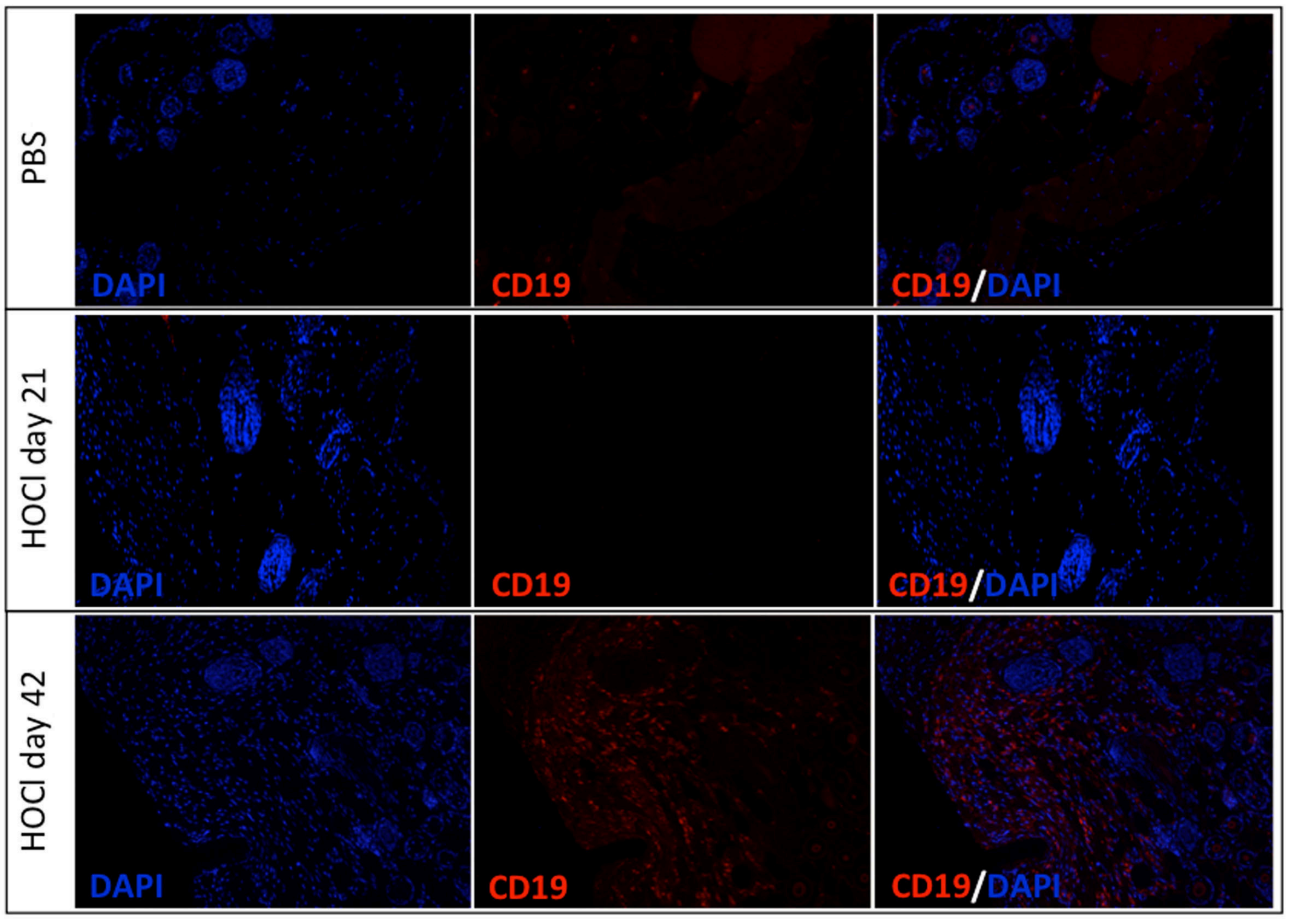
**Representative images of skin sections immunostained for CD19 in phosphate-buffered saline (PBS) and HOCl mice at day 21 and day 42**. Representative images of PBS skin (top row), HOCl skin at day 21 (middle row), and HOCl skin at day 42 (bottom row) immunostained with DAPI (left column), with anti-CD19 antibody (middle column), and with both (right column) (*n* = 5 per group).

Next we wondered if anomalies in B cell homeostasis were also observed outside the spleen and within disease-targeted organs. To do so, we assessed CD19 expression in skin samples from the two groups and found a significant B cell infiltration in HOCl skin at day 42, but no difference with PBS mice at day 21 (Figure 8).

Taken together with our previous results, these findings might indicate that B cells could be recruited from the spleen to infiltrate the skin.

### Splenic B Cell Functional Properties Are Altered in HOCl Mice

As human SSc B cells display functional anomalies that contribute to the pathogenesis of the disease, we next wondered whether the same holds true for HOCl splenic B cells. Therefore, we sought to investigate their pro-inflammatory, anti-inflammatory, and pro-fibrotic properties by studying their production of various cytokines.

#### Splenic B Cell Production of Pro-inflammatory Cytokines Is Increased in HOCl Mice

Based on data from a preliminary work (not shown), we selected two candidate proteins, IL-6 and CCL3, to assess the pro-inflammatory properties of B cells in the HOCl model.

**Figure 9:**
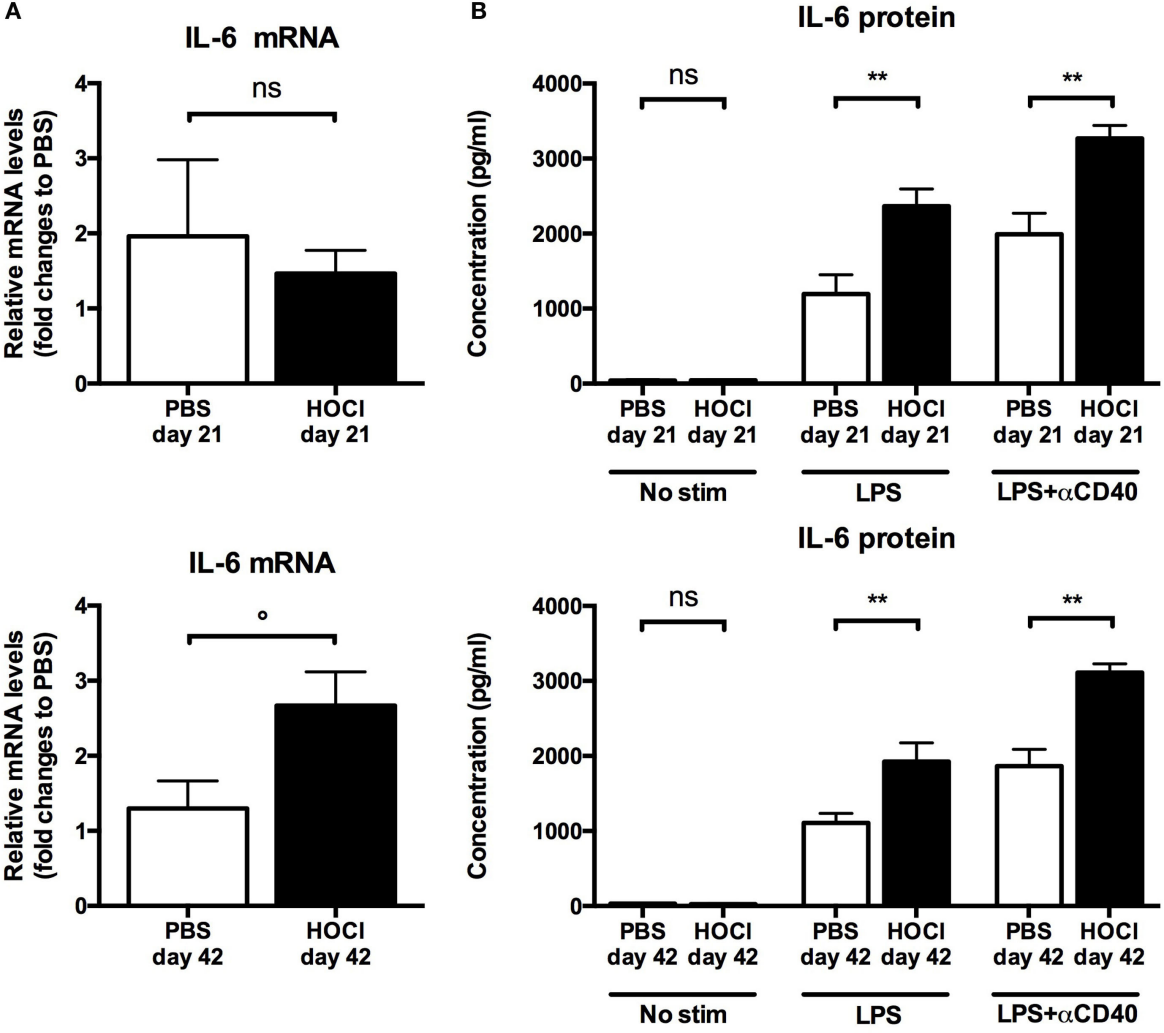
**Interleukin (IL)-6 production by splenic B cells in phosphate-buffered saline (PBS) and HOCl mice at day 21 and day 42**. **(A)** IL-6 mRNA levels in splenic B cells after collection and sorting, normalized to GUSB and expressed as fold changes to PBS day 21 for day-21 groups (top row), and to PBS day 42 for day-42 groups (bottom row) (*n* = 4–6 per group). **(B)** IL-6 supernatant levels after culture of splenic B cells for 48 h with various stimulation conditions [none, lipopolysaccharide (LPS), LPS + anti-CD40 antibody] at day 21 (top row) and day 42 (bottom row), expressed in picograms per milliliter (*n* = 6–8 per group).

**Figure 10:**
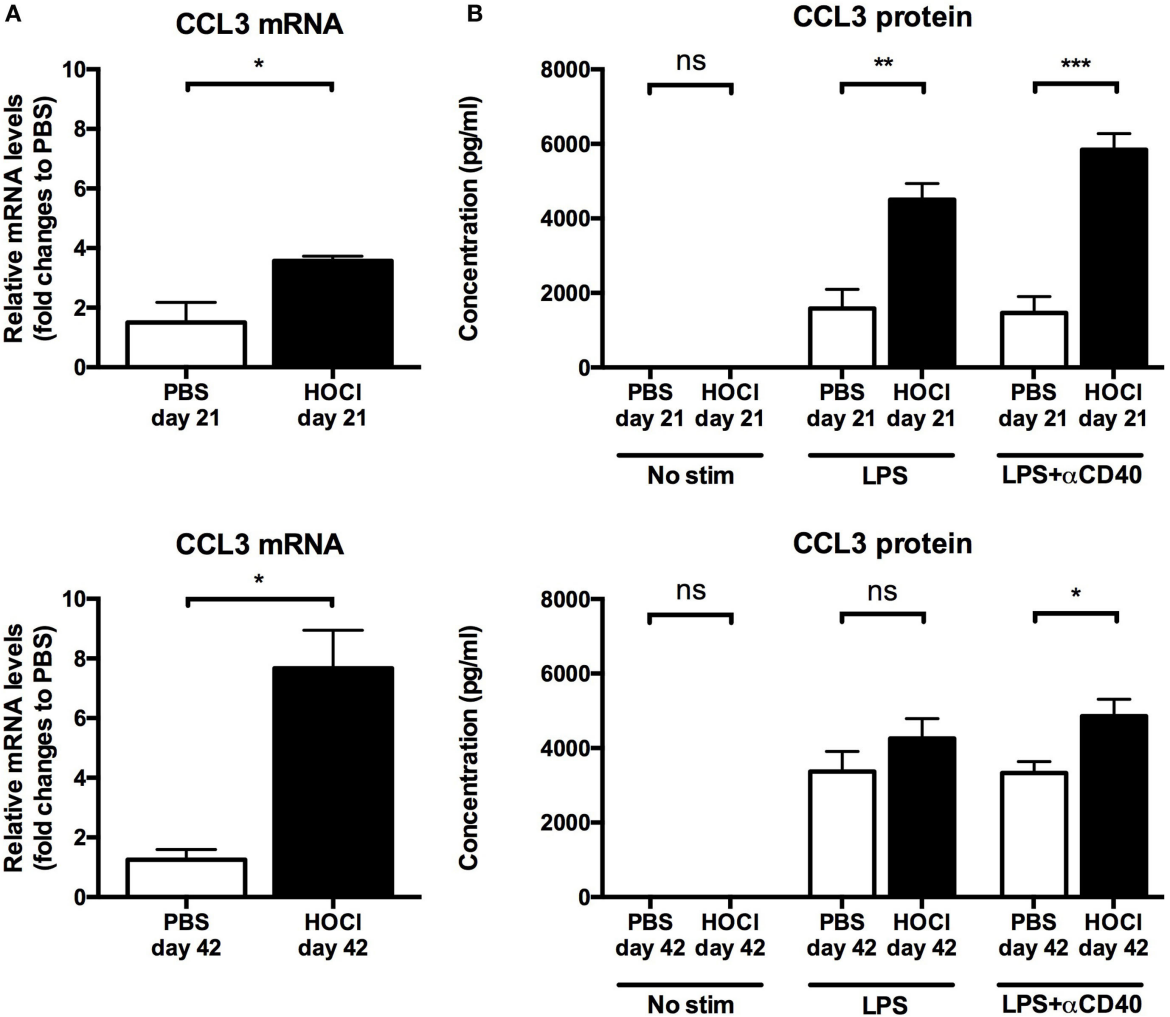
**CCL3 production by splenic B cells in phosphate-buffered saline (PBS) and HOCl mice at day 21 and day 42**. **(A)** CCL3 mRNA levels in splenic B cells after collection and sorting, normalized to GUSB and expressed as fold changes to PBS day 21 for day-21 groups (top row), and to PBS day 42 for day-42 groups (bottom row) (*n* = 4–6 per group). **(B)** CCL3 supernatant levels after culture of splenic B cells for 48 h with various stimulation conditions [none, lipopolysaccharide (LPS), LPS + anti-CD40 antibody] at day 21 (top row) and day 42 (bottom row), expressed in picograms per milliliter (*n* = 6–8 per group).

First we measured IL-6 and CCL3 mRNA levels in splenic B cells *ex vivo* (just after collection and sorting). IL-6 mRNA levels did not differ at day 21 (*p* = 0.83); but there was a trend for a significant increase in the HOCl group at day 42 (*p* = 0.06) (Figure 9A). CCL3 production was significantly higher in HOCl mice at both time points (*p* = 0.02 in both cases) (Figure 10A).

As to further explore these anomalies, we then studied the secretion of IL-6 and CCL3 in splenic B cells cultured with various stimulation conditions. Without immunostimulation, there was only negligible basal secretion of both cytokines at both time points. When stimulated by LPS alone or with anti-CD40 antibody, HOCl B cells produced more IL-6 and CCL3 than the control group at both time points (Figures 9B and 10B). This increase always reached statistical significance, except for CCL3 at day 42 with LPS stimulation (*p* = 0.18) (Figures 9B and 10B). IL-6 and CCL3 levels in HOCl B cells did not differ between day 21 and day 42 in both stimulation conditions.

Overall, these results suggested that splenic B cells display pro-inflammatory features in HOCl mice.

#### B Cell Production of IL-10 and Breg Levels Are Reduced in HOCl Mice

**Figure 11:**
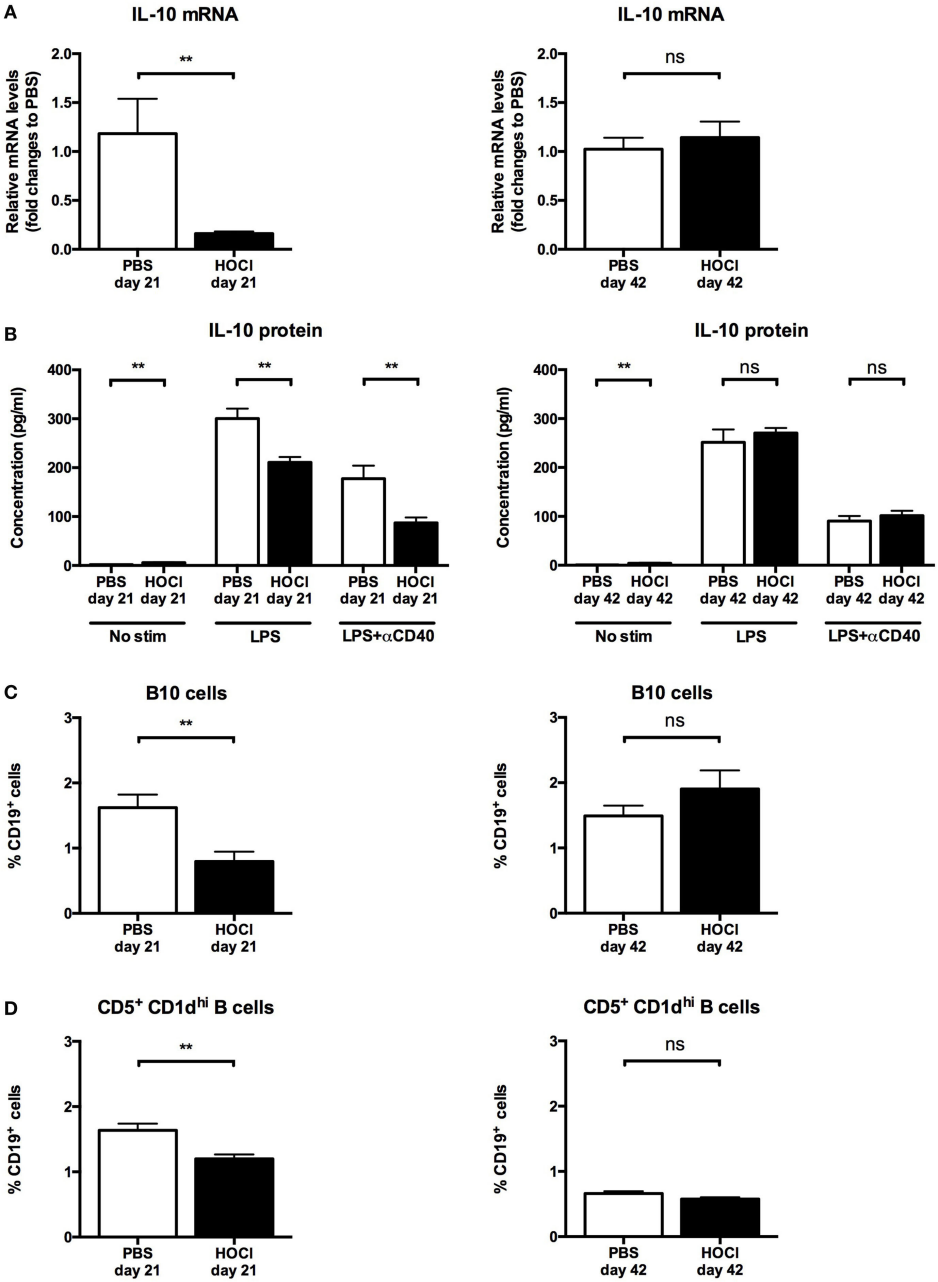
**Interleukin (IL)-10 production and regulatory B cell levels in phosphate-buffered saline (PBS) and HOCl mice at day 21 and day 42**. **(A)** IL-10 mRNA levels in splenic B cells after collection and sorting, normalized to GUSB and expressed as fold changes to PBS day 21 for day-21 groups (left column), and to PBS day 42 for day-42 groups (right column) (*n* = 4–6 per group). **(B)** IL-10 supernatant levels after culture of splenic B cells for 48 h with various stimulation conditions [none, lipopolysaccharide (LPS), LPS + anti-CD40 antibody] at day 21 (left column) and day 42 (right column), expressed in picograms per milliliter (*n* = 6–8 per group). **(C)** Proportion of B10 cells identified by intracellular IL-10 detection at day 21 (left column) and day 42 (right column), expressed as percentages of CD19^+^ cells (*n* = 10 per group). **(D)** Proportion of CD5^+^ CD1d^hi^ B cells identified by conventional membrane staining at day 21 (left column) and day 42 (right column), expressed as percentages of CD19^+^ cells (*n* = 7–8 per group).

As there is a growing body of evidence implicating B cells in immune response suppression and regulatory processes in autoimmune diseases (25), we next studied the anti-inflammatory properties of B cells in the HOCl model. We first assessed the production of IL-10, a potent anti-inflammatory cytokine, by splenic B cells. When measured *ex vivo*, IL-10 mRNA levels were significantly lower in HOCl B cells at day 21 (*p* = 0.01) but identical at day 42 (*p* = 0.82) (Figure 11A). After culture and stimulation by LPS alone or with anti-CD40 antibody, similar results were found: a significant decrease in IL-10 secretion by HOCl B cells at day 21 (*p* = 0.002 with both stimulations) and identical levels at day 42 (*p* = 0.51 with LPS and *p* = 0.47 with LPS + anti-CD40 antibody) (Figure 11B). Of note, without immunostimulation, IL-10 production by HOCl B cells was significantly higher than PBS mice but at negligible levels.

**Figure 12:**
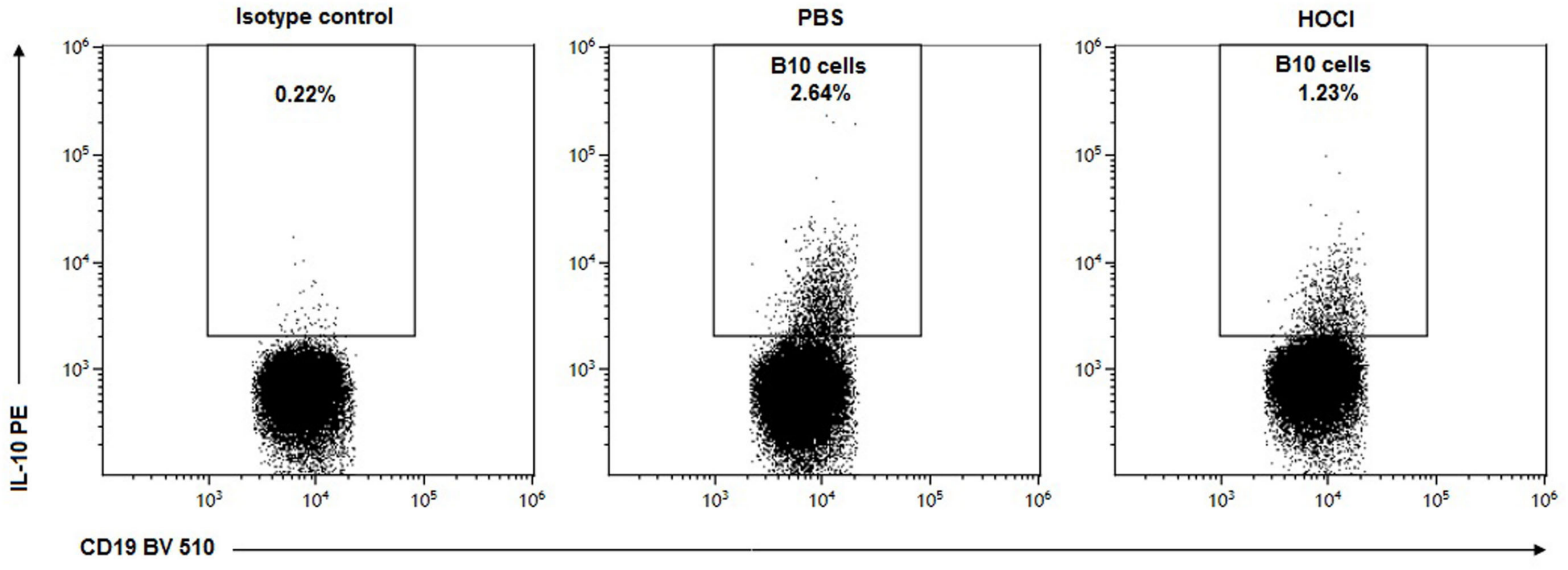
**Representative dot-plots of B10 cells in phosphate-buffered saline (PBS) and HOCl mice at day 21**. Representative CD19/interleukin (IL)-10 dot-plots gated on B cells from PBS (middle column) and HOCl (left column) mice at day 21, showing a decreased proportion of B10 cells in the HOCl mouse.

Within the B cell compartment, the main source of IL-10 production is Bregs, a recently identified subset endowed with anti-inflammatory properties (25). In mice, Bregs are formally identified as CD19^+^ IL-10^+^ cells (“B10” cells) and mostly contained within the CD19^+^ CD5^+^ CD1d^hi^ subset (24). To confirm our previous results, we next measured splenic Breg counts using both phenotypic definitions. Here again, we made similar observations: a significant decrease in B10 cells (*p* = 0.004) and in CD5^+^ CD1d^hi^ B cells (*p* = 0.006) in HOCl mice at day 21; but no difference at day 42 (*p* = 0.39 and *p* = 0.24, respectively) (Figures 11C,D and 12).

Overall, these results suggested that B cell anti-inflammatory properties are impaired at the early inflammatory stage of the experimental disease.

#### Splenic B Cells Display Pro-Fibrotic Properties in HOCl Mice

**Figure 13:**
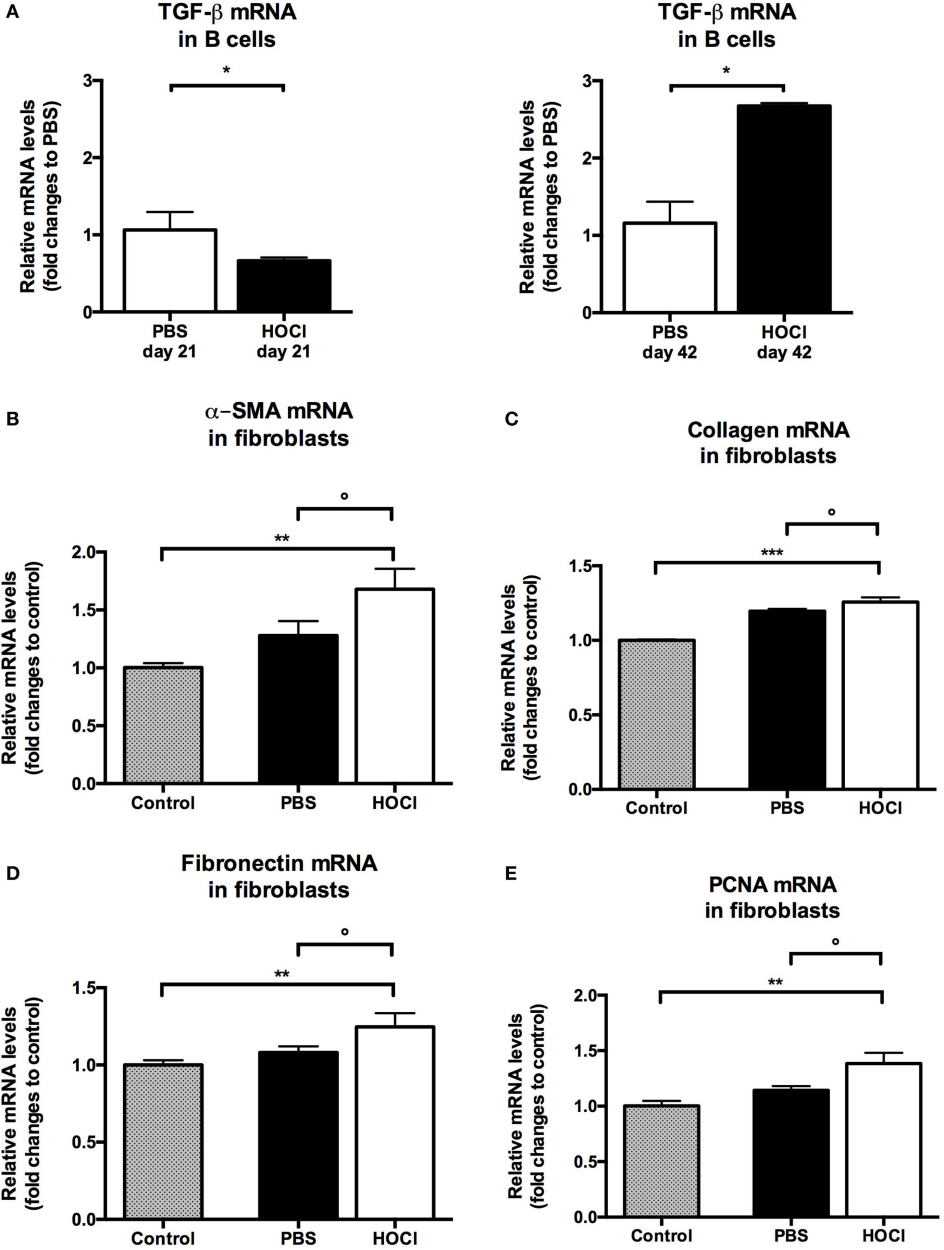
**Evaluation of pro-fibrotic properties of splenic B cells in phosphate-buffered saline (PBS) and HOCl mice at day 21 and day 42**. **(A)** Transforming growth factor (TGF)-β mRNA levels in splenic B cells after collection and sorting, normalized to GUSB and expressed as fold changes to PBS day 21 for day-21 groups (left column), and to PBS day 42 for day-42 groups (right column) (*n* = 4–6 per group). **(B–E)** mRNA levels of markers of fibrosis [α-smooth muscle actin (α-SMA) **(B)**, collagen **(C)**, and fibronectin **(D)**] and proliferation [proliferating cell nuclear antigen (PCNA) **(E)**] in 3T3 fibroblasts cultured alone (control; gray bars), with day-42 PBS B cells (white bars) or with day-42 HOCl B cells (black bars). Results normalized to GAPDH and expressed as fold changes to control (*n* = 5–8 per group).

As B cells have been shown to contribute to fibrogenesis in patients with SSc (16), we then wondered if similar findings could be made in our experimental model. We measured mRNA levels of TGF-β, a major pro-fibrotic cytokine, in HOCl and PBS splenic B cells at both time points. TGF-β production by HOCl B cells was found decreased at day 21 (*p* = 0.03) but increased at day 42 (*p* = 0.02) when compared to the control group (Figure 13A).

Finally, we next studied if B cells could directly induce a pro-fibrotic phenotype in fibroblasts. We found that fibroblasts cocultured with HOCl B cells produced higher levels of α-SMA (*p* = 0.007), collagen (*p* = 0.0008), and fibronectin (*p* = 0.009) than when cultured alone (Figures 13B–D). Coculture with HOCl B cells also triggered fibroblast proliferation as we observed a significant increase in PCNA (*p* = 0.006) (Figure 13E). These mRNA levels tended to be higher in fibroblasts cocultured with HOCl B cells than with PBS B cells (*p* = 0.08 for α-SMA, *p* = 0.09 for collagen, *p* = 0.15 for fibronectin, *p* = 0.06 for PCNA) (Figures 13B–E). Further studies are warranted to confirm these results.

Overall, these observations suggest that B cells also display pro-fibrotic properties in HOCl mice.

## Discussion

To our knowledge, this is the first study to focus on a thorough examination of B cells in an animal model of SSc. Our results can be summarized as follows: (1) HOCl mice exhibit alterations in B cell subset distribution (early expansion of transitional B cells; late expansion of the mature naive subset; and decrease in plasmablasts and memory B cells); (2) HOCl splenic B cells display enhanced pro-inflammatory features (increased production of IL-6 and CCL3); (3) HOCl splenic B cell anti-inflammatory properties are impaired at the early inflammatory stage of the experimental disease (decreased IL-10 production and Breg counts); (4) HOCl splenic B cells may contribute to fibrogenesis by producing TGF-β and inducing a pro-fibrotic phenotype in fibroblasts; and (5) these anomalies are dynamic and vary over the course of the experimental disease.

Perturbations in B cell homeostasis are well documented in human SSc (8–12) but have been rarely assessed in animal models. Aside from the present study, splenic B cell counts and subset distribution have only been examined in tight-skin (Tsk) mice, where they were found similar to controls (26). This makes our work the first to identify alterations in B cell subset homeostasis in an experimental model of SSc. More importantly, the anomalies found in HOCl mice are close to those reported in human SSc. Just as splenic B cell counts are decreased during the early active stage of the experimental protocol, circulating B cell lymphopenia is associated with disease activity and severity in SSc patients (27, 28). Characteristic B cell subset modifications in human SSc consist in an expansion of mature naive B cells and a depletion in memory B cells and plasmablasts (8–12), all of which were observed in HOCl mice in our study. Overall, it appears that the HOCl model correctly approximates the perturbations in B cell homeostasis observed in human SSc. These splenic anomalies could also bear a pathophysiological relevance, as they are associated with a B cell infiltration in the skin at the late stage.

B cell functional anomalies, and especially abnormal cytokine production, have been reported in SSc patients and experimental models (9–12, 14, 16, 26, 29–31). In both contexts, B cells display enhanced pro-inflammatory and pro-fibrotic properties, as well as impaired immune suppression capabilities, which pleads for a significant role in the pathogenesis of the disease and makes them a relevant therapeutic target. HOCl splenic B cells were found to produce higher levels of IL-6 than PBS mice. This confirms results from previous works that also documented an increased B cell secretion of IL-6 in this model (32), in the Tsk and bleomycin-induced SSc models (26, 29–31) and in human SSc (14). B cell overproduction of IL-6 is a significant feature of SSc pathogenesis, since treatment by rituximab induces a decrease in both IL-6 serum levels and skin fibrosis in SSc patients (13). Our study confirms that the HOCl model replicates this important characteristic of SSc B cells. Besides IL-6, we observe in our study a B cell overproduction of CCL3, a chemokine that promotes macrophage migration within inflammatory sites, which has never been described either in SSc models or patients. Interestingly, CCL3 levels are increased in the serum and bronchoalveolar fluid of SSc patients and associated with alveolitis and interstitial lung disease (33–35). As activated CD11b^+^ macrophages are present in the skin of HOCl mice (36), one could hypothesize that production of CCL3 by B cells may participate to their recruitment and activation in lesioned sites. Interestingly, B cell overproduction of IL-6 and CCL3 is documented not only at the early inflammatory stage, but also at the late fibrotic stage of the experimental disease. As sustained inflammation ultimately leads to fibrogenesis, this could indicate a dual role of these cytokines over time (initially pro-inflammatory and eventually pro-fibrotic).

A crucial result of our work is the documentation of impaired anti-inflammatory properties in HOCl B cells at the early stage of the protocol. Using multiple methods, we found a decrease in B cell production of IL-10 and a reduction in Breg counts in HOCl spleens. Similar observations were made in human SSc (9–11), which makes it yet another feature of SSc B cells adequately reproduced in this model. Interestingly, circulating Breg counts are inversely correlated with C-reactive protein serum levels, suggesting that, just like in HOCl mice, reduction in Breg counts occurs during the inflammatory stage of the disease (11). Bregs are currently the subject of intense research, as their therapeutic administration in several animal models of autoimmune diseases yielded promising results (25). Their potential as a treatment option in SSc remains, however, to be explored.

Aside from their role in inflammation, several studies suggested that B cells also participate in fibrogenesis during SSc. In this work, we found an increased B cell production of TGF-β at the late stage, confirming previous results in this model (32) and in others (30, 31). In SSc patients, B cells are able to induce production of extracellular matrix components by dermal fibroblasts (16). Preliminary data presented in this work tend to suggest that a similar phenomenon occurs in HOCl mice, but further studies are warranted to confirm this observation. Interestingly, we also observed a decreased TGF-β production by B cells at the early stage. Aside from its potent pro-fibrotic effect, TGF-β is also involved in immune response regulation (37). This result could suggest pleiotropic effects for TGF-β in this model, depending on the stage of the experimental disease.

Overall, our results suggest that B cells could participate in the early inflammatory events of the experimental protocol through an overproduction of pro-inflammatory cytokines (IL-6 and CCL3) and an impairment of their anti-inflammatory capabilities (decreased production of IL-10 and TGF-β, reduced levels of Bregs); and in the late fibrotic events through a cytokine overproduction (TGF-β and IL-6) which may induce a pro-fibrotic phenotype in fibroblasts. These functional anomalies are close to those encountered in human SSc.

Our work has some limitations. Most of our results are descriptive in nature, which does not allow for a definitive assessment of their relevance in the pathogenesis of the experimental disease. Further studies, either functional and/or mechanistical in design (such as complete B cell or specific subset depletions), are warranted to draw definitive conclusions.

In conclusion, this work reports, for the first time in an SSc model, the existence of anomalies in B cell homeostasis and functional properties that approximate those displayed by SSc patients. Furthermore, it suggests that these anomalies vary over the course of the disease, which has never been considered before and may contribute to the inflammatory and fibrotic events observed in SSc. This makes the HOCl mouse a relevant experimental model for the study of B cells, and especially B cell-targeted therapies, in SSc.

## Author Contributions

The authors confirm that all individuals listed as authors met all the required criteria for authorship (substantial contributions to the conception or design of the work; or the acquisition, analysis, or interpretation of data for the work; drafting the work or revising it critically for important intellectual content; final approval of the version to be published; and agreement to be accountable for all aspects of the work in ensuring that questions related to the accuracy or integrity of any part of the work are appropriately investigated and resolved).

## Conflict of Interest Statement

The authors declare that the research was conducted in the absence of any commercial or financial relationships that could be construed as a potential conflict of interest. The reviewer HD and handling Editor declared their shared affiliation, and the handling Editor states that the process nevertheless met the standards of a fair and objective review.
