# Supplementary material for: B Cell Homeostasis and Functional Properties Are Altered in an Hypochlorous Acid-Induced Murine Model of Systemic Sclerosis
Source: Front Immunol. 2017 Feb 7;8:53. doi: 10.3389/fimmu.2017.00053 (PMC5293837; doi:10.3389/fimmu.2017.00053)
Supplement: Supplementary file 1 [file Table_1.PDF]

Supplementary Material

B cell homeostasis and functional properties are altered in an hypochlorous acid-induced murine model of systemic sclerosis

Sébastien Sanges[1,2,3,4], Manel Jendoubi[1,2], Niloufar Kavian[5], Carine Hauspie[1,2,6], Silvia Speca[1,2], Jean-Charles Crave[7], Thomas Guerrier[1,2], Guillaume Lefèvre[1,2,3,4,6], Vincent Sobanski[1,2,3,4], Ariel Savina[8], Eric Hachulla[1,2,3,4], Pierre-Yves Hatron[1,2,3,4], Myriam Labalette[1,2,6], Frédéric Batteux[5], Sylvain Dubucquoi[1,2,6], David Launay[1,2,3,4,*]

[1]Univ. Lille, U995 - LIRIC - Lille Inflammation Research International Center, Lille, France

[2]INSERM, U995, Lille, France

[3]CHU Lille, Département de Médecine Interne et Immunologie Clinique, Lille, France

[4]Centre National de Référence Maladies Systémiques et Auto-immunes Rares (Sclérodermie Systémique), Lille, France

[5]Université Paris Descartes, Sorbonne Paris-Cité, Faculté de Médecine, Institut Cochin INSERM U1016 et Laboratoire d'immunologie biologique, AP-HP Hôpital Cochin, 75679 Paris cedex 14, France

[6]CHU Lille, Institut d'Immunologie, Lille, France

[7]Octapharma France SAS, Medical department, 62 bis avenue Andre Morizet, 92100 Boulogne-Billancourt

[8]Institut Roche, Boulogne Billancourt, France

*** Correspondence:**
David Launay
david.launay@univ-lille2.fr

1 Supplementary Table 1. Primers used in qPCR experiments

**Oligomer**	**Sequence**
**PRIMERS USED FOR SKIN SAMPLES AND FIBROBLASTS**	
Col1a1-F	5’- GAG TAC TGG ATC GAC CCT AAC CAA - 3’
Col1a1-R	5’-ACA CAG GTC TGA CCT GTC TCC AT- 3’
alpha-SMA-F	5’-CCT GAC GGG CAG GTG ATC- 3’
alpha-SMA-R	5’-ATG AAA GAT GGC TGG AAG AGA GTC T- 3’
Fibronectin-F	5’- GATGCTCCCACTAACCTCCA-3’
Fibronectin-R	5’-CGGTCAGTCGGTATCCTGTT-3’
PCNA-F	5’-TCGGGTGAATTTGCACGTATAT- 3’
PCNA-R	5’-CCCCATTCTTTGCACAGGAT- 3’
IL1b-F	5’-AGCTCTCCACCTCAATGGAC- 3’
IL1B-R	5’-AGGCCACAGGTATTTTGTCG- 3’
IL6-F	5’-CAGAATTGCCATTGCACAAC- 3’
IL6-R	5’-ACTGGCAAAAGGATGGTGAC- 3’
TGFb1-F	5’-CCC GAA GCG GAC TAC TAT GCT- 3’
TGFb1-R	5’-GTT TTC TCA TAG ATG GCG TTG TTG- 3’
TNFa-F	5’-CCACCACGCTCTTCTGTCTA- 3’
TNFa-R	5’-GAGGCCATTTGGGAACTTCT- 3’
GAPDH-F	5’-ATG GGA AGC TTG TCA TCA ACG- 3’
GAPDH-R	5’-GGC AGT GAT GGC ATG GAC TG- 3’
**PRIMERS USED FOR B CELLS**	
IL6-F	5’- GTTCTCTGGGAAATCGTGGA - 3’
IL6-R	5’- CAGAATTGCCATTGCACAAC - 3’
IL10-F	5’- CTTAATGCAGGACTTTAAGGGTTA - 3’
IL10-R	5’-ATTCATGGCCTTGTAGACACC - 3’
CCL3-F	5’-CCAGCCAGGTGTCATTTTCC-3’
CCL3-R	5’-AGGCATTCAGTTCCAGGTCA -3’
TGFb1-F	5’-AGAGACGTGGGGACTTCTTG - 3’
TGFb1 -R	5’-GAATAGGGGCGTCTGAGGAA - 3’
Gusb-F	5’-CAGAGCGAGTATGGAGCAGA-3’
Gusb -R	5’-CGTCATGAAGTCGGCGAAAT - 3’

alpha-SMA: α-smooth muscle actin; CCL: CC-chemokine ligand; Col1a1: collagen 1, α1 chain; F: forward; GAPDH: glyceraldehyde 3-phosphate dehydrogenase; Gusb: β-glucuronidase; IL: interleukin; PCNA: proliferating cell nuclear antigen; R: reverse; TGFb: transforming growth factor β; TNFa: tumor necrosis factor α.
