# Supplementary material for: B Cell Homeostasis and Functional Properties Are Altered in an Hypochlorous Acid-Induced Murine Model of Systemic Sclerosis
Source: Front Immunol. 2017 Feb 7;8:53. doi: 10.3389/fimmu.2017.00053 (PMC5293837; doi:10.3389/fimmu.2017.00053)
Supplement: Supplementary file 2 [file Table_2.PDF]

Supplementary Material

B cell homeostasis and functional properties are altered in an hypochlorous acid-induced murine model of systemic sclerosis

Sébastien Sanges[1,2,3,4], Manel Jendoubi[1,2], Niloufar Kavian[5], Carine Hauspie[1,2,6], Silvia Speca[1,2], Jean-Charles Crave[7], Thomas Guerrier[1,2], Guillaume Lefèvre[1,2,3,4,6], Vincent Sobanski[1,2,3,4], Ariel Savina[8], Eric Hachulla[1,2,3,4], Pierre-Yves Hatron[1,2,3,4], Myriam Labalette[1,2,6], Frédéric Batteux[5], Sylvain Dubucquoi[1,2,6], David Launay[1,2,3,4,*]

[1]Univ. Lille, U995 - LIRIC - Lille Inflammation Research International Center, Lille, France

[2]INSERM, U995, Lille, France

[3]CHU Lille, Département de Médecine Interne et Immunologie Clinique, Lille, France

[4]Centre National de Référence Maladies Systémiques et Auto-immunes Rares (Sclérodermie Systémique), Lille, France

[5]Université Paris Descartes, Sorbonne Paris-Cité, Faculté de Médecine, Institut Cochin INSERM U1016 et Laboratoire d'immunologie biologique, AP-HP Hôpital Cochin, 75679 Paris cedex 14, France

[6]CHU Lille, Institut d'Immunologie, Lille, France

[7]Octapharma France SAS, Medical department, 62 bis avenue Andre Morizet, 92100 Boulogne-Billancourt

[8]Institut Roche, Boulogne Billancourt, France

*** Correspondence:**
David Launay
david.launay@univ-lille2.fr

1 **Supplemental Table 2. Antibodies used in flow cytometry experiments.**

Antigen	Fluorochrome	Isotype	Source	Clone	Dilution	Supplier	Antibody Reference	Isotype Control Reference
**PANEL "SPLEEN CELL SUBSETS"**								
CD19	Brilliant Violet 510	IgG2a	Rat	1D3	1/100	BD Biosciences	562956	562952
CD3	PC7	IgG2b	Rat	17A2	1/200	BioLegend	100219	400617
CD4	PE	IgG2a	Rat	H129.19	1/100	BD Biosciences	553653	551799
CD8	Pacific Blue	IgG2a	Rat	53-6,7	1/100	BD Biosciences	558106	558109
CD11b	APC-C7	IgG2b	Rat	M1/70	1/200	BD Biosciences	557657	552773
CD335	APC	IgG2a	Rat	29A1.4	1/50	BioLegend	137608	400511
**PANEL "B2 CELLS"**								
CD19	Brilliant Violet 510	IgG2a	Rat	1D3	1/100	BD Biosciences	562956	562952
CD93	FITC	IgG2b	Rat	AA4.1	1/100	BD Biosciences	559156	553988
CD23	Pacific Blue	IgG2a	Rat	B3B4	1/200	BioLegend	101616	400527
IgM	PC7	IgG2a	Rat	II/41	1/100	eBiosciences	25-5790	25-4321
CD21	APC	IgG2a	Rat	7E9	1/400	BioLegend	123411	400511
B220	PE	IgG2a	Rat	RA3-6B2	1/200	BioLegend	103207	400507
**PANEL "B1 CELLS"**								
CD19	Brilliant Violet 510	IgG2a	Rat	1D3	1/100	BD Biosciences	562956	562952
CD5	FITC	IgG2a	Rat	53-7.3	1/200	BioLegend	100605	400505
CD23	Pacific Blue	IgG2a	Rat	B3B4	1/200	BioLegend	101616	400527
IgM	APC	IgG2a	Rat	II/41	1/200	BD Biosciences	550676	553932
B220	PE	IgG2a	Rat	RA3-6B2	1/200	BD Biosciences	103208	400507
CD43	PC7	IgG2a	Rat	S7	1/200	BD Biosciences	562866	552784
**PANEL "GC & ASC"**								
CD19	BV510	IgG2a	Rat	1D3	1/100	BD Biosciences	562956	562952
CD38	Pacific Blue	IgG2a	Rat	90	1/200	BioLegend	102719	400527
GL7	PE	IgM	Rat	GL7	1/100	BioLegend	144607	400807
CxCR4	APC	IgG2b	Rat	2B11	1/100	eBiosciences	17-9991	17-9991
CD138	PC7	IgG2a	Rat	281-2	1/200	BioLegend	142513	400522

CD22	FITC	IgG1	Rat	OX-97	1/100	BioLegend	126105	400405
**PANEL "MEMORY B CELLS"**								
CD19	Brilliant Violet 510	IgG2a	Rat	1D3	1/100	BD Biosciences	562956	562952
CD93	FITC	IgG2b	Rat	AA4.1	1/100	BD Biosciences	559156	553988
CD38	Pacific Blue	IgG2a	Rat	90	1/200	BioLegend	102719	400527
IgM	PC7	IgG2a	Rat	II/41	1/100	eBiosciences	25-5790	25-4321
**PANEL "REGULATORY B CELLS"**								
CD19	Brilliant Violet 510	IgG2a	Rat	1D3	1/100	BD Biosciences	562956	562952
CD5	FITC	IgG2a	Rat	53-7,3	1/200	BioLegend	100605	400505
CD1d	Alexa Fluor 647	IgG2b	Rat	1B1	1/200	BioLegend	123511	400626

CXCR: CXC-chemokine receptor; Ig: immunoglobulin; IL:interleuki
